# Electricity as an Anæsthetic

**Published:** 1860-10

**Authors:** 


*Electricity as an Anæsthetic.*—A part of a very large work upon Electro Therapeutics has been devoted to the above theme, in which the author concludes it is of comparatively little value, generally producing one pain instead of another; that if the electric current is strong enough, the patient cannot bear the pain it produces, if not strong enough, then it is useless, and in fact it is worse than useless in any way it can be used, as a local anæsthetic. The current is so rapidly conveyed through the body, that it can only act instantaneously as a local application, and is consequently ineffectual to a part. B.

